# Letter from the Editor in Chief

**DOI:** 10.19102/icrm.2024.15067

**Published:** 2024-06-15

**Authors:** Moussa Mansour


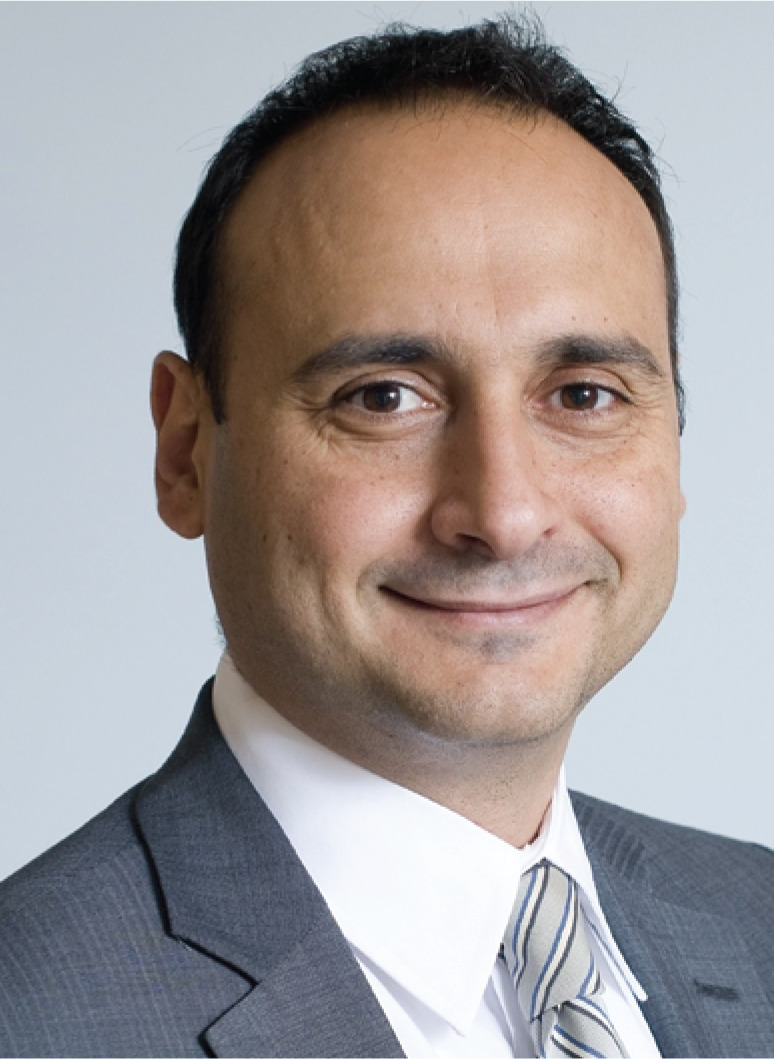


Dear readers,

The annual scientific meeting of the Heart Rhythm Society took place in Boston in May. As expected, there was a strong focus on pulsed-field ablation (PFA) for atrial fibrillation (AF). This topic was covered in core curriculum lectures, abstracts, live case presentations, and late-breaking clinical trials.

Among all late-breaking clinical trials on PFA, I would like to highlight two studies that I believe will have a significant impact on the practice of AF ablation. The first presentation was on the long-term safety and effectiveness of PFA for paroxysmal AF using data from the Assessment of Safety and Effectiveness in Treatment Management of Atrial Fibrillation with the Biosense Webster IRE Ablation System (admIRE) multicenter trial, presented by Vivek Reddy.^1^ The admIRE trial is the Investigational Device Exemption study for the VARIPULSE™ variable loop circular PFA catheter from Biosense Webster. It enrolled 291 patients with paroxysmal AF and met its primary safety and efficacy endpoints. Protocol-defined primary adverse events occurred in 2.9% of the patients. During 12 months of follow-up, the primary effectiveness success was 74.6% (95% confidence interval, 69.1%–80.1%). The procedure time was short at 81 min, and 25% of cases were performed without fluoroscopy.

The second study that I want to highlight is the Treatment of Persistent Atrial Fibrillation with Sphere-9 Catheter and Affera Mapping and Ablation System (SPHERE Per-AF) study, presented by Elad Anter, which investigated the safety and efficacy of a lattice tip dual-energy ablation catheter from Medtronic (Minneapolis, MN, USA).^2^ This is the first randomized study to compare a dual-energy radiofrequency–PFA catheter with a conventional radiofrequency catheter for persistent AF. The study enrolled 420 patients and met its primary endpoint of non-inferiority. The primary effectiveness endpoint, which included freedom from arrhythmia recurrence, was observed for 73.8% and 65.8% of patients in the investigational and control arms, respectively (*P* < .0001 for non-inferiority).^2^ Major procedural or device-related complications occurred in three patients in the investigational arm and in two patients in the control arm (*P* < .0001 for non-inferiority).^2^ Procedural times were shorter in the investigational arm compared to those in the control arm (*P* < .0001).

I believe that both of these studies provide additional confirmation of the superiority of PFA over thermal ablation and will accelerate the adoption of this novel energy source for AF ablation.

On a personal note, I would like to use this opportunity to announce that I will be stepping down as the Editor-in-Chief of *The Journal of Innovations in Cardiac Rhythm Management*. During the past 10 years, we achieved significant accomplishments, which include indexing in Scopus/Embase in 2019 and PubMed in 2020. We also witnessed a 60% increase in submission of research manuscript submissions. None of these successes could have happened without the expertise of the journal’s editorial team members, who are among the most devoted, organized, and proficient people I have ever worked with. It was a true pleasure and privilege to work with them and enjoy their partnership, and I feel proud and fulfilled when considering the educational material that we all strived to provide to you during this period. Together, we were also able to successfully guide the journal through the coronavirus disease 2019 pandemic despite the unique real-world challenges faced.

In stepping down, I am also pleased to introduce Dr. Devi Nair as the next Editor-in-Chief of the journal. Dr. Nair is the Director of the Cardiac Electrophysiology Division at St. Bernard’s Heart & Vascular Center in Jonesboro, Arkansas, and a Clinical Adjunct Professor at the University of Arkansas for Medical Sciences. She is an extremely accomplished clinical investigator and has led many landmark clinical studies in the field of cardiac electrophysiology. In addition to being a physician–scientist, Dr. Nair is a practicing electrophysiologist with a busy clinical practice, and, as such, I believe that she will continue to focus on the practical aspect of clinical cardiac electrophysiology in the journal educational material. I am confident that the journal will continue to flourish under her leadership.

I would like to thank the scientific community, who has trusted us with their studies and science and submitted their work for publication. More importantly, I want to thank you, the readers, for your support and commitment to reading the journal.


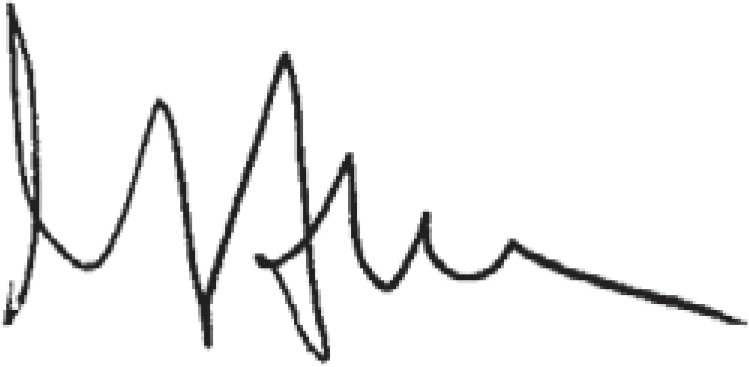


Best personal regards,

Moussa Mansour, md, fhrs, facc

Editor in Chief

*The Journal of Innovations in Cardiac Rhythm Management*

MMansour@InnovationsInCRM.com

Clinical Director, Demoulas Center for Cardiac Arrhythmias

Director, Atrial Fibrillation Program

Jeremy Ruskin and Dan Starks Endowed Chair in Cardiology

Massachusetts General Hospital

Boston, MA 02114
